# Singleton molecular species delimitation based on COI-5P barcode sequences revealed high cryptic/undescribed diversity for Chinese katydids (Orthoptera: Tettigoniidae)

**DOI:** 10.1186/s12862-019-1404-5

**Published:** 2019-03-14

**Authors:** Zhijun Zhou, Huifang Guo, Li Han, Jinyan Chai, Xuting Che, Fuming Shi

**Affiliations:** Key Laboratory of Invertebrate Systematics and Application of Hebei Province, College of Life Sciences, Hebei University, Baoding, 071002 Hebei China

**Keywords:** Katydids, DNA barcoding, Species delimitation, Cryptic species, Clustering-based method, Similarity-based method

## Abstract

**Background:**

DNA barcoding has been developed as a useful tool for species discrimination. Several sequence-based species delimitation methods, such as Barcode Index Number (BIN), REfined Single Linkage (RESL), Automatic Barcode Gap Discovery (ABGD), a Java program uses an explicit, determinate algorithm to define Molecular Operational Taxonomic Unit (jMOTU), Generalized Mixed Yule Coalescent (GMYC), and Bayesian implementation of the Poisson Tree Processes model (bPTP), were used. Our aim was to estimate Chinese katydid biodiversity using standard DNA barcode cytochrome c oxidase subunit I (COI-5P) sequences.

**Results:**

Detection of a barcoding gap by similarity-based analyses and clustering-base analyses indicated that 131 identified morphological species (morphospecies) were assigned to 196 BINs and were divided into four categories: (i) MATCH (83/131 = 64.89%), morphospecies were a perfect match between morphospecies and BINs (including 61 concordant BINs and 22 singleton BINs); (ii) MERGE (14/131 = 10.69%), morphospecies shared its unique BIN with other species; (iii) SPLIT (33/131 = 25.19%, when 22 singleton species were excluded, it rose to 33/109 = 30.28%), morphospecies were placed in more than one BIN; (iv) MIXTURE (4/131 = 5.34%), morphospecies showed a more complex partition involving both a merge and a split. Neighbor-joining (NJ) analyses showed that nearly all BINs and most morphospecies formed monophyletic cluster with little variation. The molecular operational taxonomic units (MOTUs) were defined considering only the more inclusive clades found by at least four of seven species delimitation methods. Our results robustly supported 61 of 109 (55.96%) morphospecies represented by more than one specimen, 159 of 213 (74.65%) concordant BINs, and 3 of 8 (37.5%) discordant BINs.

**Conclusions:**

Molecular species delimitation analyses generated a larger number of MOTUs compared with morphospecies. If these MOTU splits are proven to be true, Chinese katydids probably contain a seemingly large proportion of cryptic/undescribed taxa. Future amplification of additional molecular markers, particularly from the nuclear DNA, may be especially useful for specimens that were identified here as problematic taxa.

**Electronic supplementary material:**

The online version of this article (10.1186/s12862-019-1404-5) contains supplementary material, which is available to authorized users.

## Introduction

Taxonomic ambiguities and uncertainties are frequently generated due to cryptic or hidden species [1]. Species identification based on morphological characters requires experienced taxonomists [2]. Recently, DNA barcode have been recommended for the insect biodiversity evaluation [3]. DNA barcoding employs a single or a few standardized, highly variable and easily amplified DNA fragments for species identification [4, 5]. The 5′ portion of mitochondrial cytochrome c oxidase subunit I (COI-5P) has become the standard insect barcoding marker. Numerous studies rely on COI-5P as the only molecular information for insect species delimitation and identification [6–9]. DNA barcodes not only substantially improve the accuracy of species identifications but also accelerate the study of taxonomically difficult and hyperdiverse taxon. The introduction of DNA barcoding as an auxiliary method in taxonomy has many benefits. Firstly, DNA barcodes can lead to an easy assignment of specimens of certain life stages (e.g. eggs, larvae, nymphs or pupae) to known species [10]. Second, DNA barcoding requires a solid taxonomic background to use as a reference [11]. DNA barcodes can also add scientific value to standard museum specimens, as the information they contain is revealed through molecular analyses [12]. Third, DNA barcodes can accelerate the discovery of cryptic/undescribed species and have been incorporated into the new species description for several zoological groups [13–16].

Recently, several similarity-based species delimitation approaches, e.g. Barcode Index Number (BIN), REfined Single Linkage algorithm (RESL), Automatic Barcode Gap Discovery (ABGD) and a Java program uses an explicit, determinate algorithm to define Molecular Operational Taxonomic Unit (jMOTU); and clustering-based approaches, e.g. Generalized Mixed Yule Coalescent (GMYC), Bayesian implementation of the Poisson Tree Processes model (bPTP) have highlighted extensive inconsistency in morphological taxonomy [17, 18]. ABGD analysis generate MOTUs based on features in sequence distance distributions [19]. RESL employs single linkage clustering as a tool for the preliminary assignment of records into one MOTU [20]. BIN system developed within the Barcode of Life Data (BOLD, www.barcodinglife.org) system to register the OTUs delineated by RESL [20]. jMOTU, a Java program uses an explicit, determinate algorithm to define MOTU [21]. GMYC and bPTP based on quite different models. GMYC applies single or multiple time thresholds to delimit species in a Maximum likelihood context, using ultrametric trees [22, 23]. bPTP is similar to GMYC, but used substitution calibrated trees [24]. Either single approach may lead to over- and/or underestimating species diversity. Here, we carried out different approaches for species delineation using DNA sequence data in order to have more robust results. The distinction between intraspecific and interspecific genetic divergence is critical for DNA barcoding; greater intraspecific divergence produces a greater likelihood of overlap with interspecific divergence [11]. For similarity-based approaches, the distance cut-off used for the determination of MOTUs is important, but arbitrary. Even no one threshold captures all species concepts or operational criteria [12]. Relaxed clustering-based methods that permit larger divergences within cohesive clusters may give even greater utility to similarity-based approaches [25]. The choice of a species delimitation method from molecular data has a considerable effect on estimated species entities and, thus, also on species richness estimates [26]. BOLD system [27] provides a unique environment for sharing data across projects; it not only supports all phases of the analytical pathway, from specimen collection to a tightly validated barcode library, but has already integrated many analysis tools. The BOLD system assigns a BIN for all barcode records. The BIN system will also help us to focus on those taxa that share the same BIN or split amongst multiple BINs [28].

Single-locus species delimitation methods have become popular due to the adoption of the DNA barcoding paradigm [17]. When delimiting putative species based on single-locus data, researchers should consider using both clustering- and similarity-based methods to account for the shortcomings of different methods [17, 29, 30]. Previously reported cases of high-failure rates in using traditional morphospecies definitions were largely resolved upon using MOTUs instead of traditionally described morphospecies, which suggested that some morphospecies may require taxonomic revision [25].

DNA barcoding has been used to document both grasshoppers [10, 31–34] and katydids [35, 36]. Unfortunately, these studies included only a few species and a limited number of specimens from each species, which prevents the rigorous assessment of species boundaries among closely related lineages and for calculation of intraspecific distances. Recently, Hawlitschek et al. (2017) presented a large-scale DNA barcode data set that includes 748 COI sequences from 127 species of Central European crickets, katydids and grasshoppers [37].

The Katydid diversity is rich but woefully underexplored in China. Both *Mecopoda elongata* and *Gampsocleis gratiosa* have a long history as singing pets in China. Researchers have reported that numerous species belong to the family Tettigoniidae Krauss, 1902 [38], but only a very limited COI-5P barcode records were available in the GenBank and BOLD systems. Barcode-based species identification relies on a comparison of its DNA barcode with those of determined individuals. To be effective, species-level assignments require a reference sequences database which represents all known species [39].

As different methods may yield inconformity conclusions [40], the accurate species identification and/or delimitation requires further integrative analysis. This study represents the first large-scale barcoding study of the family Tettigoniidae. Different molecular species delimitation methods such as BIN, RESL, jMOTU, ABGD, GMYC, and bPTP were applied to an unexplored Chinese katydid fauna. The main aims were (i) to present the largest species-level barcoding study for the Chinese katydids to date and then characterize the range of genetic divergence; (ii) to evaluate the correspondence between the identified morphospecies and the defined barcode groupings using molecular species delimitation methods; (iii) to infer species diversity and compare it to traditionally identified morphospecies sorting; and (iv) to test for the existence of a hidden diversity among otherwise well-defined taxa. Here, we define independently barcode groupings that remain morphologically indistinguishable from each other as cryptic or hidden species.

## Materials and methods

### Specimen collection and morphological identification

We collected 2576 katydid specimens throughout China. Specimens were fixed in absolute ethanol and were transferred to − 20 °C storage prior to genomic DNA extraction. Whenever possible, more than one location was sampled for each species to survey for intraspecific variation. Due to the polymorphism and remarkably wide distribution, a broader sampling (*n* ≥ 10 specimens) was particularly intended for 39 morphospecies, namely, *Conanalus axinus*, *Conanalus pieli*, *Conocephalus bambusanus*, *Conocephalus gladiatus*, *Conocephalus longipennis*, *Conocephalus maculatus*, *Conocephalus melaenus*, *Deflorita deflorita*, *Ducetia japonica*, *Ducetia spina*, *Elimaea cheni*, *Euconocephalus pallidus*, *Euxiphidiopsis capricercus*, *Gampsocleis gratiosa*, *Gampsocleis sedakovii*, *Gampsocleis sinensis*, *Gampsocleis ussuriensis*, *Hemielimaea chinensis*, *Hexacentrus japonicas*, *Hexacentrus unicolor*, *Isopsera denticulate*, *Kuwayamaea brachyptera*, *Mecopoda niponensis*, *Orthelimaea trapzialis*, *Parapsyra nigrovittata*, *Parapsyra notabilis*, *Phaneroptera falcate*, *Pseudorhynchus concisus*, *Ruidocollaris truncatolobata*, *Ruspolia dubia*, *Ruspolia jezoensis*, *Ruspolia lineosa*, *Sinochlora szechwanensis*, *Tettigonia chinensis*, *Xizicus fascipes*, *Xizicus howardi*, *Xizicus kweichowensis*, *Xizicus spathulatus*, and *Xizicus szechwanensis*.

Specimens were sorted morphologically and were taxonomically identified at least to the subfamily-level. For ease of information management, all unidentified specimens without a scientific name were assigned to an interim species (hereafter noted as BIN-species) by BINs provided by BOLD systems, and were noted by the generic/subfamily name plus sp.1, sp.2 and so on. For example, *Atlanticus* spp. were assigned to 19 BINs, so we noted them as *Atlanticus* sp1, *Atlanticus* sp2, *Atlanticus* sp3, etc. The specimen described as ‘undescribed genus’ were identified up to the subfamily level. All voucher specimens were preserved at the College of Life Sciences, Hebei University. More precise taxonomic determinations have been added for some specimens since their initial identification, and further taxonomic detail will be added to the BOLD systems as work progresses after publication.

### DNA extraction, amplification and sequencing

DNA extraction from the leg muscle tissue of each specimen was carried out using the TIANamp Genomic DNA Kit (Tiangen Biotech, Beijing, China), following the manufacturer’s instructions. The universal primer pairs LCO1490/HCO2198 [41] were used to amplify and sequence the animal barcode region. PCRs were performed with 96-well plates. The reaction master mix consisted of 2400 μL 2 × Premix Taq™, 480 μL each primer (10 μmol/L) and 1152 μL water. This mixture was prepared for each plate, and each well contained 1 × Premix Taq™, 1 μmol each primer, 3~5 ng genomic DNA. The PCR profile was comprised of an initial denaturation step of 2 min at 95 °C, and 35 cycles of 30 s at 94 °C, 40 s at 50 °C and 1 min at 72 °C, with a final extension of 7 min at 72 °C.

Amplicons were checked through a 1% agarose gel and bi-directional sequencing was performed at GENEWIZ (Tianjin, China). Sequences were manually edited and assembled into a consensus sequence using SeqMan Pro [42]. Consensus sequences, specimen collection data, specimen images and sequence trace files were uploaded to the Barcode of Life Data System (BOLD) and are available to the public domain as part of the project DNA Barcoding to Katydids from China (DBKC).

### Data analysis

We constructed our primary dataset from BOLD systems, including all public records that were geographically limited in China, and with a length ≥ 600 bp. To reduce computational requirements, we divided our entire dataset into four subsets by a subfamily or consonant subfamilies based on the four monophyletic lineages that were recognized by our previous mitogenomic Bayesian inference analysis with the site-heterogeneous CAT-GTR model [43]. The DS-DBCHL dataset is composed of 596 barcode sequences from Conocephalinae, Hexacentrinae, and Lipotactinae. The DS-DBMEC dataset is composed of 376 barcode sequences from Meconematinae. The DS-DBPPM dataset is composed of 993 barcode sequences from Phaneropterinae, Pseudophyllinae, and Mecopodinae. The DS-DBTB dataset is composed of 200 barcode sequences from Tettigoniinae and Bradyporinae.

### Sequence analysis module of BOLD systems

BIN was used as a registry for the records on the BOLD systems [27], which provided a means of confirming the concordance between barcode sequence clusters and species designations [20, 28]. Cluster sequence analysis using RESL was independent of the BIN registry of BOLD systems [20]. Genetic distances were calculated and summarized using the “Distance Summary” and “Barcode Gap Analysis” tools on BOLD systems [27]. Barcode gap analysis provides the distribution of distances within each species and the distance to the nearest neighbor (NN) of each species. Species are tested for the presence of the barcode gap. The NN distance is the genetic distance between a species and its closest congeneric relative. All sequences (> 600 bp) were aligned using MUSCLE [44], phylogeny model used was the Kimura 2-parameter (K2P) [45], and pairwise deletion of missing data was done. The correlation between the maximum intraspecific variation (K2P) against record count and maximum geographic extent (km) of sampled individuals was determined for each species sampled from more than one sites.

### Similarity-based methods: jMOTUs and ABGD

In performing the additional “Sequence analysis module” of BOLD, we also applied two similarity-based methods to generate MOTUs. Each of the four datasets was analyzed with jMOTU_define 2.04 [21, 46] using different cut-offs (from 0 to 25 bp). ABGD analysis was performed to sort the sequences into hypothetical species that are based on the barcode gap, which can be observed whenever the divergence among organisms that belonging to the same species is smaller than the divergence among organisms from different species [19].

### Clustering-based methods: GMYC and bPTP

Previous studies suggest that an additional bias may be introduced for clustering-based methods when duplicate haplotypes are not removed [17]. Prior to species delimitation analyses with two clustering-based methods, we applied DAMBE [47] to remove duplicate haplotypes. A total of 530 unique haplotypes from the DS-DBPPM dataset, 147 haplotypes from DS-DBTB, 158 haplotypes from DS-DBMEC, and 390 haplotypes from DS-DBCHL were included in the further analyses. Ultrametric trees were estimated with BEAST v1.8.3 [48] using a Yule speciation prior and an uncorrelated lognormal relaxed clock. The best-fitting substitution models were selected under the Bayesian Information Criteria (BIC), as was implemented in jModelTest 2.1.7 [49]. Each of the four datasets was analyzed for 200 million iterations with the first 10% discarded as burn-in. Posterior probabilities (PP) were estimated under a sampling frequency of every 10,000 steps. Tracer v.1.6 (http://tree.bio.ed.ac.uk/software/tracer/) was used to determine when the analyses became stable and to check whether the effective sample size (ESS) values were greater than 200, as recommended by Drummond et al. (2007). The consensus trees obtained before the Markov chain reached stable and convergent likelihood values were discarded as burn-in with TreeAnnotator v.1.7. The resulting ultrametric trees were used for both single-threshold GMYC (sGMYC) [22] and multiple-threshold GMYC (mGMYC) [23] analyses using the Splits [50] and Ape [51] libraries.

The coalescent clustering-based method (bPTP) was performed using the online server (http://species.h-its.org/) and the Bayesian Inference trees from MrBayes 3.2 [52]. We ran bPTP analyses for 500,000 MCMC generations with a thinning of 500 and a burn-in of 0.1. Convergence of the MCMC chain was assessed as recommended by Zhang et al. (2013). Outgroups were pruned before conducting bPTP analyses to avoid bias that may arise if some of the outgroup taxa were too distantly related to the ingroup taxa [24].

### Comparison morphospecies and MOTU of species delimitation method outputs

All NJ-K2P trees of unique COI-5P haplotypes were performed using MEGA v7.0 [53]. The results of the species delimitation methods were summarized on the NJ-K2P trees with a midpoint root. Four different taxonomic scenarios between morphospecies (equated with BIN-species for unidentified specimens) and MOTU clustering methods outputs can be distinguished: (i) ‘MATCH’, whereby the members of a species were placed in one MOTU that had no other members; (ii) ‘MERGE’, whereby the members of a species were placed in one MOTU together with members from another species; (iii) ‘SPLIT’, whereby the members of a species were assigned to more than one MOTU that had no other specimens from another species; and (iv) ‘MIXTURE’, whereby each species show a more complex partition involving both ‘MERGE’ and ‘SPLIT’. To further compare the results of different species delimitation methods, we also employed the adjusted Wallace coefficients analysis [54] to quantify MOTUs agreement with Linnaean species labels or among MOTUs from different species delimitation methods through the website Comparing Partitions (http://www.comparingpartitions.info/) [55]. Here, we excluded singletons and only discussed species or MOTUs that are represented by more than one specimen. Finally, MOTUs were defined considering only the clades represent groups of barcodes recovered in at least four of the seven species delimitation methods [20].

## Results

DNA was extracted from 2576 Chinese katydid specimens, of which 2131 specimens (82.73%) were successfully sequenced for COI-5P barcode. All records were removed that less than 600 bp, contained contaminants, had stop codons, flagged as misidentifications or errors. In summary, we generated 2131 original COI-5P sequences from 131 morphospecies, including 528 specimens that were identified to genus level and one specimen that was placed at the subfamily level. The remaining unidentified lineages were represented using BINs, because they were either unable to be reliably identified based on the available reference materials or they were still undescribed. Additional 34 published COI-5P barcode sequences (Additional file 1: Table S1) were retrieved from GenBank in the BOLD system and included for further analyses. The entire dataset containing 2165 Chinese katydids COI-5P barcode sequences comprised 1225 distinct haplotypes, and represented 60 genera, 9 subfamilies of the family Tettigoniidae Krauss, 1902, including Bradyporinae (*n* = 1), Conocephalinae (*n* = 490), Hexacentrinae (*n* = 102), Lipotactinae (*n* = 4), Meconematinae (*n* = 376), Mecopodinae (*n* = 53), Phaneropterinae (*n* = 861), Pseudophyllinae (*n* = 79), and Tettigoniinae (*n* = 199). Nearly a third of the morphospecies (39/131 = 29.77%) included 10 or more specimens. The number of barcode sequences per species varied from 1 up to 142 in the commonly occurring *Ducetia japonica*, dispersed throughout China.

### Intra- and interspecific genetic divergences

The intra- and interspecific genetic divergences within different taxonomic ranks are detailed in Table 1. The intraspecific divergence for 109 morphospecies represented by more than one specimen averaged 1.54% (ranging from 0 to 27.45%). However, the intraspecific divergence for 77 unidentified BIN-species represented by more than one specimen averaged 0.39% (ranging from 0 to 2.81%). The identified morphospecies, which were assigned to more than one BIN, were the major cause of higher intraspecificity. The mean interspecific divergence for identified morphospecies and unidentified BIN-species at genus level were 15.35% (ranging from 0 to 28.74%) and 12.86% (ranging from 1.07 to 28.07%), respectively. The mean interspecific divergence for identified morphospecies and unidentified BIN-species at family level were 22.29% (ranging from 2.16 to 32.88%) and 21.67% (ranging from 3.27 to 32.78%), respectively. The normalized mean intraspecific and minimum interspecific distance were 1.40 ± 0.02 and 0% for 109 identified morphospecies, in contrast to 0.45 ± 0.01 and 1.07% for 77 unidentified BIN-species, respectively (Table 2).

**Table 1:** Summary of K2P distances between barcode sequences at each taxonomic level

	Label	n	Taxa*	Comparisons	Min Dist (%)	Mean Dist (%)	Max Dist (%)	SE Dist (%)
Entire dataset	Within Species	2070	186	40,374	0.00	1.44	27.45	0.00
	Within Genus	2091	34	88,915	0.00	14.21	28.82	0.00
	Within Family	2164	1	2,211,077	1.85	22.21	34.09	0.00
Identified Morphospecies	Within Species	1614	109	36,966	0.00	1.54	27.45	0.00
	Within Genus	1526	24	55,140	0.00	15.35	28.74	0.00
	Within Family	1636	1	1,245,324	2.16	22.29	32.88	0.00
Unidentified BIN-species	Within BIN-Species	456	77	3408	0.00	0.39	2.81	0.00
	Within Genus	502	18	13,109	1.07	12.86	28.07	0.00
	Within Family	528	1	122,611	3.27	21.67	32.78	0.00

*Singleton morphospecies or BIN-species were excluded

**Table 2:** Normalized divergence statistics

Quantity	Entire dataset	Identified Morphospecies	Unidentified BIN-species
Species Count	186	109	77
Mean Within-Species Dist (%)	1.01	1.40	0.45
SE of Mean Within-Species Dist (%)	0.01	0.02	0.01
Min Between-Species Dist (%)	0.00	0.00	1.07

The within-species distribution is normalized to reduce bias in sampling at the species level

**Fig. 1:**
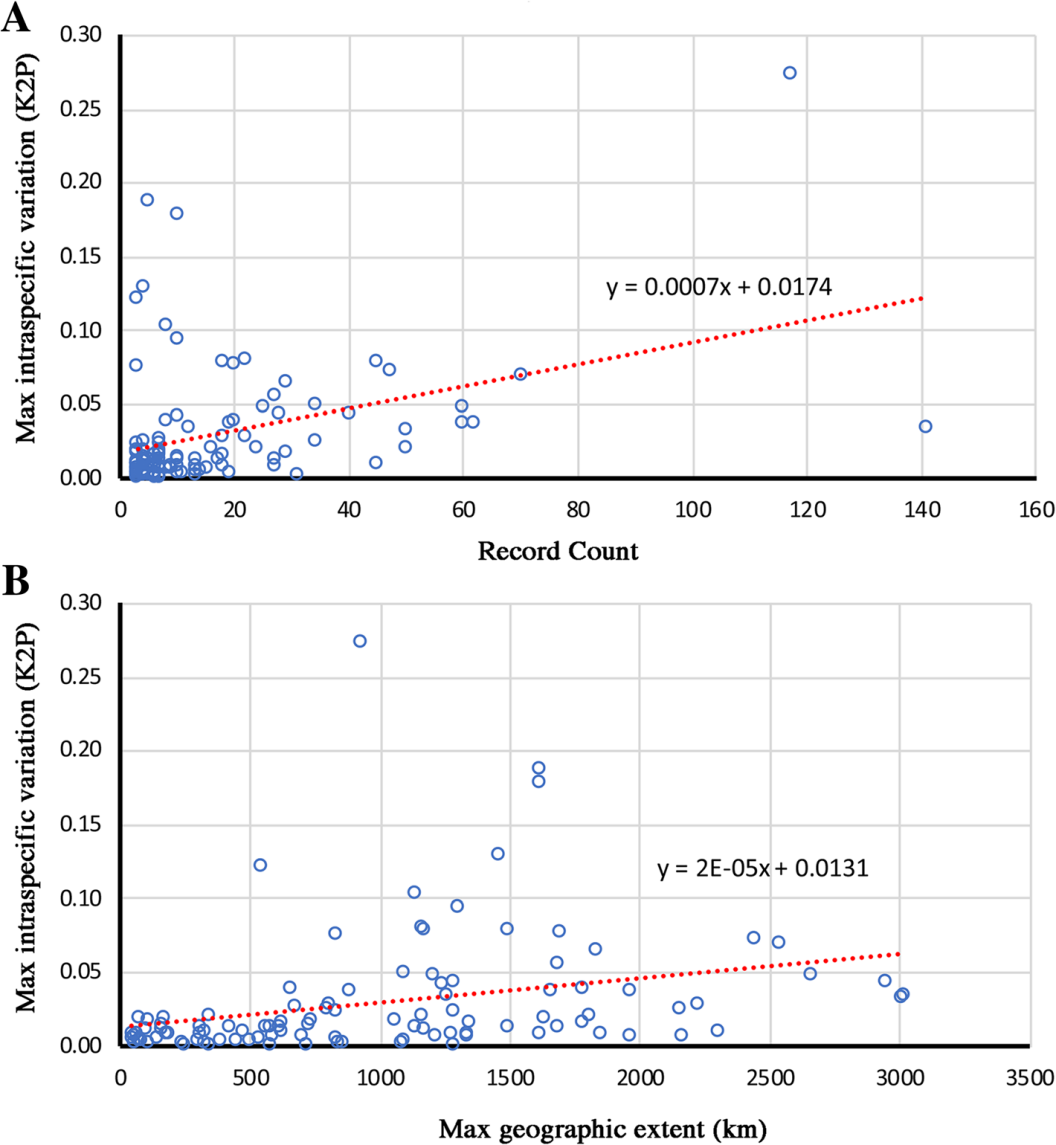
Maximum intraspecific variation (K2P) against record count (**a**) and maximum geographic extent (**b**) of sampled individuals. **a** linear regression, y = 0.0007x + 0.0174, Adjusted R-square = 0.142, *P* < 0.001; **b** linear regression, y = 2E-05x + 0.0131, Adjusted R-square = 0.080, *P* < 0.001

For the entire dataset, the distance of 22 morphospecies and 16 BIN-species to its NN was smaller than 2%, in which the distance of 16 of 22 morphospecies to NN was less than maximum intraspecific distance (Additional file 2: Table S2). Meanwhile, there are 13 morphospecies and one BIN-species, the distance to NN was larger than 2%, but less than the maximum intraspecific distance (Table 3). Deep intraspecific divergences (> 2%) overlapping with the distance to NN were detected in 21 morphospecies and one BIN-species, namely, *Conanalus robustus* (9.25%), *Conocephalus bidentatus* (18.85%), *Conocephalus longipennis* (17.81%), *Euconocephalus pallidus* (3.45%), *Euxiphidiopsis capricercus* (27.45%), *Gampsocleis gratiosa* (4.91%), *Gampsocleis sedakovii* (4.9%), *Gampsocleis ussuriensis* (3.78%), *Hexacentrus japonicus* (4.4%), *Mecopoda niponensis* (7.97%), *Phyllomimus sinicus* (8.09%), *Pseudorhynchus pyrgocoryphus* (10.36%), *Ruidocollaris truncatolobata* (6.59%), *Ruspolia dubia* (3.27%), *Ruspolia yunnana* (3.78%), *Sinochlora szechwanensis* (7.92%), *Xiphidiopsis autumnalis* (12.14%), *Xiphidiopsis bituberculata* (2.65%), *Xiphidiopsis gurneyi* (13.02%), *Xizicus spathulatus* (9.43%), *Xizicus howardi* (7.79%), and *Ruidocollaris* sp. 7 (2.81%). The linear regressions analysis indicate the maximum intraspecific variation (K2P) was significantly correlated with record count (*P* < 0.001) and maximum geographical extent of sampled individuals (*P* < 0.001), but had limited explanatory power (Adjusted R-square = 0.080 and 0.142) (Fig. 1).

**Table 3:** Highlighted records which the distance to nearest neighbor (NN) less than 2% or Max intra-specific distance

Species	Mean Intra-Sp	Max Intra-Sp	Nearest Neighbour (Process ID)	Distance to NN
*Atlanticus* sp. 17	0.72	1.08	*Atlanticus* sp. 18 (RBTC726–16)	1.85
*Atlanticus* sp. 18	1.05	1.38	*Atlanticus* sp. 17 (RBTC1171–16)	1.85
*Conanalus robustus*	5.1	9.25	*Conanalus pieli* (BOCON180–16)	7.03
*Conocephalus bidentatus*	9.45	18.85	*Conocephalus maculatus* (BOCON016–16)	18
*Conocephalus japonicus*	0.15	0.15	*Conocephalus longipennis* (BOCON043–16)	0.61
*Conocephalus longipennis*	11.58	17.81	*Conocephalus japonicus* (BOCON230–16)	0.61
*Deflorita deflorita*	0.2	0.46	*Deflorita* sp. 2 (RBTC216–16)	1.55
*Deflorita* sp. 2	0.44	1.39	*Deflorita deflorita* (RBTC286–16)	1.55
*Elimaea* sp. 21	0.3	0.3	*Elimaea* sp. 22 (RBTC911–16)	1.38
*Elimaea* sp. 22	0	0	*Elimaea* sp. 21 (RBTC473–16)	1.38
*Elimaea* sp. 29	N/A	0	*Elimaea* sp. 32 (RBTC254–16)	1.7
*Elimaea* sp. 32	N/A	0	*Elimaea* sp. 29 (RBTC1636–16)	1.7
*Elimaea* sp. 33	0.31	0.46	*Elimaea* sp. 32 (RBTC254–16)	1.86
*Euconocephalus nasutus*	1.03	1.69	*Euconocephalus pallidus* (BOCON209–16)	0.15
*Euconocephalus pallidus*	1.17	3.45	*Euconocephalus nasutus* (BOCON147–16)	0.15
*Euxiphidiopsis capricercus*	0.8	27.45	*Gampsocleis gratiosa* (GHF059–16)	2.49
*Gampsocleis gratiosa*	2.62	4.91	*Euxiphidiopsis capricercus* (HLXX121–16)	2.49
*Gampsocleis sedakovii*	1.95	4.9	*Gampsocleis ussuriensis* (GHF015–16)	0.3
*Gampsocleis sinensis*	0.86	1.85	*Gampsocleis ussuriensis* (GHF028–16)	0
*Gampsocleis ussuriensis*	1.37	3.78	*Gampsocleis sinensis* (RBTC1209–16)	0
*Hexacentrus japonicus*	2.43	4.4	*Hexacentrus mundus* (BHC044–15)	3.76
*Kuwayamaea* sp. 6	0.24	0.61	*Kuwayamaea* sp. 7 (RBTC404–16)	1.07
*Kuwayamaea* sp. 7	0.23	0.46	*Kuwayamaea* sp. 6 (RBTC1654–16)	1.07
*Mecopoda niponensis*	2.12	7.97	*Mecopoda* sp. (RBTC573–16)	4.62
*Parapsyra nigrovittata*	0.13	0.46	*Parapsyra* sp. 3 (RBTC1308–16)	1.38
Parapsyra sp. 2	0.1	0.15	*Parapsyra nigrovittata* (RBTC722–16)	1.86
Parapsyra sp. 3	0.43	0.76	*Parapsyra nigrovittata* (RBTC722–16)	1.38
*Phyllomimus sinicus*	5.49	8.09	*Phyllomimus* sp. 15 (RBTC505–16)	7.72
*Phyllomimus* sp. 10	0.15	0.46	*Phyllomimus* sp. 11 (RBTC734–16)	1.23
*Phyllomimus* sp. 11	0.15	0.3	*Phyllomimus* sp. 10 (RBTC192–16)	1.23
*Pseudorhynchus pyrgocoryphus*	2.69	10.36	*Pseudorhynchus* sp. (RBTC1420–16)	5.08
*Ruidocollaris* sp. 5	0.05	0.3	*Ruidocollaris truncatolobata* (RBTC450–16)	1.07
*Ruidocollaris* sp. 7	1.25	2.81	*Ruidocollaris* sp. 5 (RBTC1281–16)	2.79
*Ruidocollaris truncatolobata*	2.82	6.59	*Ruidocollaris* sp. 5 (RBTC1246–16)	1.07
*Ruspolia dubia*	1.33	3.27	*Ruspolia jezoensis* (BOCON110–16)	0.15
*Ruspolia jezoensis*	0.7	1.39	*Ruspolia dubia* (RBTC1880–16)	0.15
*Ruspolia liangshanensis*	0.46	0.76	*Ruspolia dubia* (RBTC1895–16)	0.15
*Ruspolia yunnana*	2.12	3.78	*Ruspolia dubia* (RBTC1812–16)	2.17
*Sinochlora szechwanensis*	1.75	7.92	*Sinochlora longifissa* (RBTC649–16)	4.74
*Xiphidiopsis autumnalis*	8.41	12.14	*Xiphidiopsis gurneyi* (HLXX060–16)	10.39
*Xiphidiopsis bituberculata*	1.21	2.65	*Xiphidiopsis minorincisus* (RBTC928–16)	1.23
*Xiphidiopsis cheni*	0.81	1.38	*Xizicus* sp. (RBTC165–16)	1.85
*Xiphidiopsis gurneyi*	9.25	13.02	*Xiphidiopsis autumnalis* (HLXX065–16)	10.39
*Xiphidiopsis minorincisus*	0.51	0.77	*Xiphidiopsis bituberculata* (HLXX092–16)	1.23
*Xizicus concavilaminus*	0.31	0.46	*Xizicus kulingensis* (HLXX155–16)	0.3
*Xizicus howardi*	4.57	7.79	*Xizicus rehni* (HLXX169–16)	1.07
*Xizicus kulingensis*	N/A	0	*Xizicus concavilaminus* (RBTC356–16)	0.3
*Xizicus laminatus*	0.88	1.7	*Xizicus tinkhami* (HLXX042–16)	0.77
*Xizicus rehni*	0.89	1.54	*Xizicus howardi* (HLXX019–16)	1.07
*Xizicus* sp.	0.06	0.3	*Xiphidiopsis cheni* (HLXX071–16)	1.85
*Xizicus spathulatus*	5.28	9.43	*Xiphidiopsis maculatus* (HLXX106–16)	7.12
*Xizicus tinkhami*	0.92	0.92	*Xizicus laminatus* (HLXX037–16)	0.77

Alignment: MUSCLE (Edgar, 2004). Filters applied: ≥ 600 bp only, exclude records contained contaminants, had stop codons, flagged as misidentifications or errors; Deletion method: Pairwise deletion. NA, not applicable

### Comparing BIN-species and morphospecies identifications

The BOLD-implemented refined single-linkage algorithm that provided the BIN assignments used a 2.2% p-distance seed threshold but then refined the groupings for individual BINs and neighboring clusters based on the level of continuity in the distribution of genetic divergences among sequences [20, 56]. In total, 1635 sequences of 131 morphospecies were assigned to 196 BINs, including 52 singleton BINs, 136 concordant BINs, and 8 discordant BINs. Only one barcode of *Ducetia japonica* RBTC523–16 was not assigned to any BIN, because it contained more than 1% Ns. It was excluded from the analyses of species delimitation except for NJ-K2P tree. These cases of discrepancy are discussed in more detail in subsequent sections. The unidentified 529 specimens without formal (binomial) names were assigned to 150 BIN-species, including 77 concordant BINs and nearly half (*n* = 73) singleton BINs (Table 4). On average, we identified a different BIN for every 6.25 specimens, and 44 of 346 BINs (12.72%) included not less than 10 specimens, in which only two BINs (BOLD:ACE7214 and ADB5001) included more than 100 specimens.

**Table 4:** MOTU number of Chinese Katydids inferred by each single-locus species delimitation methods

Quantity	DBCHL	DBMEC	DBPPM	DBTB	Total
COI-5P sequences	596	376	993	200	2165
Haplotypes	390	158	530	147	1225
Identified morphospecies/BINs/Record count	40/69/579	40/51/341	43/63/551	8/13/164	131/196/1635
Singleton BINs/Record count	11/11	20/20	19/19	2/2	52/52
Concordant BINs/Record count	55/502	27/288	44/532	10/54	136/1376
Discordant BINs/Record count	3/66	4/33	0/0	1/108	8/207
Unidentified BIN-species/Record count	8/17	5/35	118/441	19/36	150/529
Singleton BINs/Record count	2/2	4/4	57/57	10/10	73/73
Concordant BINs/Record count	6/15	1/31	61/384	9/26	77/456
Total BINs count/Record count	77/596	56/376	181/992^a^	32/200	346/2164
RESL^*^	71	56	180	42	349
jMOTU (13 bp)	61	57	169	31	318
ABGD (2.15%)	42	53	140	20	255
Single-GMYC	87	66	197	32	382
Multiple-GMYC	94	60	206	37	397
bPTP	55	56	178	23	312

Record count, the number of COI-5P sequences after quality control, ^*^Alignment: MUSCLE (Edgar, 2004). Filters applied: ≥ 600 bp only, exclude records contained contaminants, had stop codons, flagged as misidentifications or errors; Deletion method: Pairwise deletion; One COI-5P sequence without BIN records in DBPPM dataset corresponded that sequences did not fulfill with barcode compliance standards. All bPTP results were from Bayesian MCMC analyses. Results of GMYC and bPTP analyses were from genealogies based on COI-5P haplotype sequences

There are four different taxonomic scenarios between BINs and morphospecies, including MATCH, SPLIT, MERGE, or MIXTURE [20]. Approximately 64.9% (*n* = 84) of the identified morphospecies were MATCH, and 1:1 corresponded with BINs (including 63 concordant BINs and 21 singleton BINs) (Table 5). The discordances between morphospecies and BINs included the members of multiple morphospecies pooled into one BIN (MERGE), a morphospecies split into more than one BIN (SPLIT), or both (MIXTURE). We found 18 morphospecies (6 pairs, two triads) that shared BINs (Table 6), in which 14 morphospecies were MERGE and shared its unique BIN with other species. The remaining 4 morphospecies were MIXTURE, and were also assigned to an additional BIN, including *Conocephalus longipennis* (2), *Euconocephalus pallidus* (1), *Ruspolia dubia* (3), and *Xizicus howardi* (2). Excluded singleton species, with a relatively high percentage of morphospecies, were SPLIT, and were assigned to more than one BIN (34/109 = 31.19%), namely, *Conanalus pieli* (7BINs), *Conanalus robustus* (3BINs), *Conocephalus bidentatus* (3BINs), *Conocephalus longipennis* (3BINs), *Conocephalus maculatus* (11BINs), *Ducetia japonica* (3BINs), *Elimaea nautica* (3BINs), *Euconocephalus pallidus* (2BINs), *Euxiphidiopsis capricercus* (2BINs), *Euxiphidiopsis spathulata* (2BINs), *Gampsocleis carinata* (3BINs), *Gampsocleis gratiosa* (5BINs), *Hexacentrus japonicus* (4BINs), *Isopsera denticulata* (2BINs), *Kuwayamaea brachyptera* (4BINs), *Mecopoda niponensis* (4BINs), *Orthelimaea trapzialis* (2BINs), *Phyllomimus detersus* (2BINs), *Phyllomimus sinicus* (6BINs), *Pseudorhynchus pyrgocoryphus* (2BINs), *Ruidocollaris truncatolobata* (2BINs), *Ruspolia dubia* (4BINs), *Ruspolia lineosa* (2BINs), *Ruspolia yunnana* (3BINs), *Sinochlora szechwanensis* (2BINs), *Tettigonia chinensis* (2BINs), *Xiphidiopsis autumnalis* (2BINs), *Xiphidiopsis gurneyi* (4BINs), *Xizicus fascipes* (2BINs), *Xizicus howardi* (3BINs), *Xizicus kweichowensis* (2BINs), *Xizicus magnus* (3BINs), *Xizicus spathulatus* (3BINs), and *Xizicus szechwanensis* (2BINs) (Table 7).

**Table 5:** Morphospecies-BIN perfect matches in this study

Species	BIN (Record count)	Species	BIN (Record count)
*Abaxisotima macrocaudata*	BOLD:ADB5605 (1)	*Parapsyra nigrovittata*	BOLD:ADB5036 (19)
*Alloducetia bifurcata*	BOLD:ADB6076 (2)	*Parapsyra notabilis*	BOLD:ACD4991 (16)
*Anelytra compressa*	BOLD:ADB9406 (2)	*Paraxantia huangshanensis*	BOLD:ADB6578 (1)
*Chizuella bonneti*	BOLD:ACD5704 (2)	*Paraxantia parasinica*	BOLD:ADB5974 (1)
*Conanalus axinus*	BOLD:ADB6973 (18)	*Paraxantia sinica*	BOLD:ADB6970 (4)
*Conanalus brevicaudus*	BOLD:ADC2356 (3)	*Phaneroptera falcata*	BOLD:AAL2811 (13)
*Conocephalus bambusanus*	BOLD:ADB5791 (24)	*Phaneroptera nigroantennata*	BOLD:ACD4406 (2)
*Conocephalus chinensis*	BOLD:ADC0408 (7)	*Prohimerta yunnanea*	BOLD:ADB6754 (1)
*Conocephalus discolor*	BOLD:ADC0317 (3)	*Pseudokuzicus pieli*	BOLD:ACD4648 (1)
*Conocephalus emeiensis*	BOLD:ADC0398 (7)	*Pseudomacedna nigrogeniculata*	BOLD:ADB4064 (1)
*Conocephalus fuscus*	BOLD:AAR9918 (6)	*Pseudorhynchus concisus*	BOLD:ADB6233 (10)
*Conocephalus gladiatus*	BOLD:ACD8581 (34)	*Pyrgocorypha gracilis*	BOLD:ADB9362 (2)
*Conocephalus melaenus*	BOLD:ACD4634 (45)	*Pyrgocorypha parva*	BOLD:ADC0410 (5)
*Conocephalus percaudatus*	BOLD:AAR9916 (3)	*Pyrgocorypha planispina*	BOLD:ADC0545 (3)
*Decma fissa*	BOLD:ACD8224 (2)	*Qinlingea brachystylata*	BOLD:ADB4056 (1)
*Deflorita deflorita*	BOLD:ADB3725 (10)	*Ruidocollaris obscura*	BOLD:ACD5159 (2)
*Deracantha onos*	BOLD:AAI9644 (1)	*Ruspolia indicus*	BOLD:ACI0393 (7)
*Ducetia spina*	BOLD:ADB5148 (18)	*Ruspolia interruptus*	BOLD:ACH8980 (5)
*Ducetia triramosa*	BOLD:ADB5531 (2)	*Ruspolia nitidula*	BOLD:ACH9706 (6)
*Elimaea annamensis*	BOLD:ADE1944 (9)	*Sinochlora longifissa*	BOLD:ACD4415 (2)
*Elimaea cheni*	BOLD:ADB3480 (11)	*Tapiena bivittata*	BOLD:ACD5863 (1)
*Euxiphidiopsis singulus*	BOLD:ADE1953 (1)	*Tegra novaehollandiae*	BOLD:ADB5353 (7)
*Gragoryella dimorpha*	BOLD:ADE1822 (1)	*Teratura geniculata*	BOLD:ACD5305 (2)
*Hemielimaea chinensis*	BOLD:ADB3478 (17)	*Teratura megafurcula*	BOLD:ACD5306 (2)
*Hemielimaea omeishanica*	BOLD:ACD5212 (7)	*Xestophrys horvathi*	BOLD:ABY3224 (1)
*Hexacentrus expansus*	BOLD:ACX8629 (2)	*Xiphidiopsis anisolobulus*	BOLD:ADE3080 (1)
*Hexacentrus mundus*	BOLD:ACX8886 (6)	*Xiphidiopsis cheni*	BOLD:ADE0541 (6)
*Hexacentrus unicolor*	BOLD:ACD7247 (60)	*Xiphidiopsis clavata*	BOLD:ACD6661 (5)
*Isopsera furcocerca*	BOLD:ADB4481 (1)	*Xiphidiopsis elongata*	BOLD:ADE3081 (1)
*Isopsera spinosa*	BOLD:ADB5805 (3)	*Xiphidiopsis fanjingshanensis*	BOLD:ADE3990 (1)
*Isopsera sulcata*	BOLD:ACD7803 (3)	*Xiphidiopsis jinxiuensis*	BOLD:ADE1671 (3)
*Letana rubescens*	BOLD:ACD5474 (3)	*Xiphidiopsis maculatus*	BOLD:ADE3991 (1)
*Lipotactes tripyrga*	BOLD:ACD6794 (4)	*Xiphidiopsis protensus*	BOLD:ADE3283 (1)
*Meconemopsis quadrinotata*	BOLD:ADE0701 (1)	*Xizicus bilobus*	BOLD:ADE0468 (1)
*Mecopoda elongata*	BOLD:AAF0977 (7)	*Xizicus biprocerus*	BOLD:ADE1374 (3)
*Mirollia bispinosa*	BOLD:ADB4148 (4)	*Xizicus divergentis*	BOLD:ADE4028 (7)
*Nigrimacula paraquadrinotata*	BOLD:ACD6675 (2)	*Xizicus incisus*	BOLD:ACD5524 (3)
*Orophyllus montanus*	BOLD:ADB9525 (1)	*Xizicus sinuatus*	BOLD:ADE1431 (3)
*Palaeoagraecia ascenda*	BOLD:ACD8365 (7)	*Xizicus tibeticus*	BOLD:ADE2569 (2)
*Palaeoagraecia brunnea*	BOLD:ADB5364 (7)	*Xizicus transversus*	BOLD:ADE2568 (2)
*Paraducetia paracruciata*	BOLD:ADB5358 (3)	*Xizicus xiai*	BOLD:ADE2811 (3)
*Parapsyra midcarina*	BOLD:ADB5037 (9)		

Number of barcodes included in each BIN was given in brackets

**Table 6:** Discordance BINs report for different morphospecies were assigned to one BIN

URI	Rank	Species 1 (Record count)	Species 2 (Record count)	Species 3 (Record count)
BOLD:AAY1322	Species	*Gampsocleis sinensis* (29)	*G. sedakovii* (60)	*G. ussuriensis* (19)
BOLD:ACD5539	Species	*Xizicus howardi* (6)	*X. rehni* (4)	
BOLD:ACD6726	Species	*Euconocephalus pallidus* (2)	*E. nasutus* (6)	
BOLD:ACD8335	Species	*Conocephalus longipennis* (3)	*C. japonicus* (2)	
BOLD:ADB3332	Species	*Xizicus kulingensis* (1)	*X. concavilaminus* (3)	
BOLD:ADB3697	Species	*Xiphidiopsis bituberculata* (7)	*X. minorincisus* (3)	
BOLD:ADB5868	Species	*Xizicus tinkhami* (2)	*X. laminatus* (7)	
BOLD:ADE4977	Species	*Ruspolia dubia* (38)	*R. jezoensis* (10)	*R. liangshanensis* (5)

The underlined species split into more than one BIN

**Table 7:** Summary of the 37 morphospecies were assigned to more than one BIN

Species	BIN (Record count)
*Conanalus pieli*	BOLD:ACD4962 (4), BOLD:ADB5725 (19), BOLD:ADB5876 (7), BOLD:ACD4960 (1), BOLD:ADB5726 (1), BOLD:ADB9877 (1), BOLD:ADC0465 (1)
*Conanalus robustus*	BOLD:ADB9302 (3), BOLD:ADB9301 (1), BOLD:ADB9303 (1)
*Conocephalus bidentatus*	BOLD:ADB9596 (2), BOLD:ADB6577 (2), BOLD:ADC0531 (1)
*Conocephalus longipennis*	BOLD:ADC0256 (3), BOLD:ADC0257 (4), BOLD:ACD8335 (3)
*Conocephalus maculatus*	BOLD:ADB6782 (2), BOLD:ADB6356 (24), BOLD:ADB5579 (2), BOLD:ACD2116 (3), BOLD:ADB6002 (5), BOLD:ACN8107 (7), BOLD:ADB6842 (5), BOLD:ACD4542 (12), BOLD:ACD4543 (6), BOLD:ADC0188 (3), BOLD:ABV1952 (1)
*Ducetia japonica*	BOLD:ACE7214 (101), BOLD:ADB6191 (7), BOLD:ACD7324 (33)
*Elimaea nautica*	BOLD:ACA6035 (2), BOLD:ADM8991 (1), BOLD:ADM8992 (1)
*Euconocephalus pallidus*	BOLD:AAP6087 (10), BOLD:ACD6726 (2)
*Euxiphidiopsis capricercus*	BOLD:ADB5001 (116), BOLD:ADE2467 (1)
*Euxiphidiopsis spathulata*	BOLD:ADB5352 (2), BOLD:ADB5870 (1)
*Gampsocleis carinata*	BOLD:ADA6038 (2), BOLD:ADA6039 (3), BOLD:ADA6037 (3)
*Gampsocleis gratiosa*	BOLD:ADA5568 (5), BOLD:ADA6837 (6), BOLD:ADA6838 (8), BOLD:ADA6839 (5), BOLD:ADA6836 (1)
*Hexacentrus japonicus*	BOLD:ACX8110 (7), BOLD:ACD8277 (14), BOLD:ACD8278 (3), BOLD:ADM2486 (4)
*Isopsera denticulata*	BOLD:ACD5194 (30), BOLD:ACD5193 (10)
*Kuwayamaea brachyptera*	BOLD:ACD7465 (14), BOLD:ADB5963 (2), BOLD:ADB3600 (5), BOLD:ADB5962 (1)
*Mecopoda niponensis*	BOLD:ACD8152 (42), BOLD:ACQ5648 (1), BOLD:ACQ0048 (1), BOLD:ACQ0049 (1)
*Orthelimaea trapzialis*	BOLD:ADB5808 (4), BOLD:ADB5615 (12)
*Phyllomimus detersus*	BOLD:ADB4776 (3), BOLD:ADB5035 (1)
*Phyllomimus sinicus*	BOLD:ADB5607 (7), BOLD:ADB5606 (2), BOLD:ACD4881 (5), BOLD:ADB4615 (6), BOLD:ADB5608 (1), BOLD:ADB6880 (1)
*Pseudorhynchus pyrgocoryphus*	BOLD:ADB7056 (7), BOLD:ADB9469 (1)
*Ruidocollaris truncatolobata*	BOLD:ACD7529 (9), BOLD:ACD6433 (20)
*Ruspolia dubia*	BOLD:ACD5503 (6), BOLD:ADE5391 (5), BOLD:ADE5392 (1), BOLD:ADE4977 (38)
*Ruspolia lineosa*	BOLD:ACD5257 (41), BOLD:ACD5256 (7)
*Ruspolia yunnana*	BOLD:ACH8981 (2), BOLD:ADE5243 (2), BOLD:ADC2408 (5)
*Sinochlora szechwanensis*	BOLD:ADB3789 (42), BOLD:ACD8228 (3)
*Tettigonia chinensis*	BOLD:ACD6622 (17), BOLD:ACD6623 (3)
*Xiphidiopsis autumnalis*	BOLD:ADE1666 (2), BOLD:ADE1667 (1)
*Xiphidiopsis gurneyi*	BOLD:ADB7052 (1), BOLD:ADE1670 (1), BOLD:ADE1669 (1), BOLD:ADE1668 (1)
*Xizicus fascipes*	BOLD:ACD4254 (7), BOLD:ADE3375 (3)
*Xizicus howardi*	BOLD:ADB5688 (10), BOLD:ADE3141 (4), BOLD:ACD5539 (6)
*Xizicus kweichowensis*	BOLD:ADB3846 (61), BOLD:ADE2939 (1)
*Xizicus magnus*	BOLD:ADE2449 (2), BOLD:ADE2447 (1), BOLD:ADE2448 (1)
*Xizicus spathulatus*	BOLD:ADE0562 (5), BOLD:ADE0560 (4), BOLD:ADE0561 (1)
*Xizicus szechwanensis*	BOLD:ADB3348 (24), BOLD:ADE0823 (3)

The underlined BINs were shared by more than one morphospecies. Number of barcodes included in each BIN was given in brackets

### Monophyletic morphospecies or BIN-species recovered by NJ-K2P trees

The NJ-K2P trees based on COI-5P haplotypes are shown in Additional files 3, 4, 5 and 6. The BIN 2.2% seed threshold was calibrated against morphological species using a selected groups of taxa: bees, butterflies and moths, fish, and birds [20]. The DBCHL dataset included 596 COI-5P barcode sequences, and was assigned to 77 BINs, including 13 singleton BINs, 61 concordant BINs, and 3 discordant BINs. NJ analysis with 390 COI-5P haplotypes sequences showed that all BIN-species represented by more than one specimen formed a monophyletic clade, except for BOLD:ACH8981, ACN8107 and ADE4977 (Additional file 3). The 579 sequences represented 40 identified morphospecies, in which 39 species included more than one specimen. 30 identified morphospecies formed monophyletic clusters. In addition, three specimens of *Conanalus brevicaudus* shared a unique COI-5P haplotype. The members of *Ruspolia dubia*, *R. jezoensis*, and *R. liangshanensis* were grouped jointly and formed a larger monophyletic clade with a low divergence (Additional file 3). The BOLD:ADE4977 includes a triad of species, including all members of *Ruspolia jezoensis* (*n* = 10), *R. liangshanensis* (*n* = 5) and most of the *R. dubia* (*n* = 38). The remaining members of *Ruspolia dubia* were assigned three BINs, BOLD:ADE5391 (*n* = 5), ADE5392 (*n* = 1), and ACD5503 (*n* = 6). The members of *Euconocephalus pallidus* were split into two closely related clades (Additional file 3), and clade B1 was formed by two specimens of *E. pallidus* and all specimens of *E. nasutus*. The singleton *Pseudorhynchus* sp. is nested within the *P. pyrgocoryphus* clade (Additional file 3). *Conanalus robustus* was split into two relatively distant clades (Additional file 3). Clade D1 corresponded to BOLD:ADB9302, and the members of clade D2 with high divergences were assigned to two BINs (BOLD:ADB9301 and ADB9303). Two specimens identified as *Conocephalus japonicus* is nested within the *C. longlpennis* clade (Additional file 3). The widely distributed morphospecies *Ruspolia lineosa* was monophyletic but contained two deeply subclusters. Almost all species delimitation analyses suggested that *R. lineosa* split into two putative species, *R. lineosa* BOLD:ACD5256 and ACD5257. Only ABGD suggested that *R. lineosa* to be a distinct species. Both sGMYC and mGMYC subsplit *R. lineosa* BOLD:ACD5257 into two parts.

The DBMEC dataset included 376 COI-5P barcode sequences and was assigned to 56 BINs, which included 24 singleton BINs, 28 concordant BINs, and 4 discordant BINs. NJ analysis with 158 COI-5P haplotypes sequences showed that all BIN-species with more than one sequence formed a monophyletic clade (Additional file 4). The 341 sequences represented 40 identified morphospecies, in which 29 species included more than one specimen. 22 identified morphospecies revealed nonoverlapping monophyletic clusters. The remaining 7 morphospecies were not monophyletic. *Euxiphidiopsis capricercus* was split into two reciprocally monophyletic clusters (Additional file 4) and corresponded to two BINs (BOLD:ADB5001 and ADE2467). Both *Xiphidiopsis gurneyi* and *X. autumnalis* were split into two reciprocally monophyletic clusters, and four clusters were grouped jointly as a separate clade (Additional file 4). The singleton species identified as *Xiphidiopsis maculatus* was embedded into the *Xizicus spathulatus* clade (Additional file 4). The members of *Xizicus howardi* were recovered in three reciprocally monophyletic clusters (Additional file 4) and corresponded to three BINs (BOLD:ACD5539, ADB5688 and ADE3141). *Xizicus concavilaminus* and *X. kulingensis* were grouped jointly (Additional file 4), and shared a single BIN (BOLD:ADB3332). *Xizicus tinkhami* and *X. laminatus* were grouped jointly (Additional file 4) and shared a single BIN (BOLD:ADB5868). *Xiphidiopsis bituberculata* and *X. minorincisus* were grouped jointly (Additional file 4) and shared a single BIN (BOLD:ADB3697). Due to their small size, the active dispersal abilities of Meconematinae katydids were highly limited.

The DBPPM dataset included 993 COI-5P barcode sequences and was assigned to 181 BINs, which included 76 singleton BINs, and 105 concordant BINs. Only one barcode (*Ducetia japonica* RBTC523–16) without BIN records corresponded to sequences that did not fulfill the barcode compliance standards. NJ analysis with 530 COI-5P haplotypes sequences showed that all BIN-species with more than one sequence formed a monophyletic clade (Additional file 5). The 551 sequences represented 43 identified morphospecies, in which 33 species included more than one specimen. Twenty-seven identified morphospecies were revealed in nonoverlapping monophyletic clusters. *Ruidocollaris truncatolobata* was split into two relatively distant clusters (Additional file 5) and corresponded to two BINs (BOLD:ACD6433 and ACD7529). One specimen that was identified as *Mecopoda* sp. is nested within the clade of *M. niponensis* (Additional file 5). *Sinochlora szechwanensis* was split into two relatively distant clades (Additional file 5). Clade C1 was formed exclusively by specimens from Yuexi, Anhui and was closely related to the species *Sinochlora longifissa*. Clade C2 was formed by the remaining specimens, and was closely related to *Sinochlora* sp2 (BOLD:ADB3463). Two specimens that were identified as *Phyllomimus* sp14 (BOLD:ADB3808) and *Phyllomimus* sp15 (BOLD:ADB6425) are nested within the *P. sinicus* clade (Additional file 5).

The DBTB dataset included 200 COI-5P barcode sequences, and was assigned to 32 BINs, which included 12 singleton BINs, 19 concordant BINs, and 1 discordant BINs. NJ analysis with 147 COI-5P haplotypes sequences showed that all BIN-species with more than one sequence formed a monophyletic clade, except for BOLD: ADA6837 and ADB3445 (Additional file 6). A total of 8 identified morphospecies were represented by 164 sequences, including 7 species that were represented by more than one specimen. The NJ analysis based on K2P distances revealed nonoverlapping clusters for 4 identified morphospecies, *Chizuella bonneti*, *Gampsocleis carinata*, *G. gratiosa*, and *Tettigonia chinensis*. In contrast, *Gampsocleis sedakovii*, *G. sinensis*, and *G. ussuriensis* were grouped jointly. The remaining 36 sequences were provisionally assigned into 19 putative species based on BINs.

### Concordance among MOTUs from similarity-based species delimitation methods

Because of its strong taxonomic performance and speed, RESL was adopted to generate MOTUs for the barcode sequences on BOLD systems [27]. The results of RESL analyses generated 349 MOTUs, which had only small discrepancies in comparison with the BINs (Table 4). For the DBCHL dataset, RESL analysis generated 71 MOTUs. The differences between BINs and RESL were that (i) four BINs (BOLD:ADE4977 representing *Ruspolia dubia*, *R. jezoensis*, *R. liangshanensis*, and ADE5391, ADE5392, ACD5503 representing *R. dubia*) were pooled in one MOTU; (ii) two BINs (BOLD:ACH8981 and ADE5243 representing *Ruspolia yunnana*) were pooled in one MOTU; (iii) two BINs (BOLD:ACD6726 representing *Euconocephalus pallidus* and *E. nasutus*, and BOLD:AAP6087 representing *E. pallidus*) were pooled in one MOTU. For the DBMEC dataset, RESL analysis generated 56 MOTUs. For the DBPPM dataset, RESL analysis generated 180 MOTUs. *Hemielimaea omeishanica* (BOLD:ACD5212) were split into two MOTUs. *Ruidocollaris* sp7 (BOLD:ADB6075) were split into four MOTUs. Meanwhile, RESL recovered *Ducetia japonica* as one MOTU, which was assigned to three BINs (BOLD:ACD7324, ADB6191, ACE7214). Two singleton BINs, BOLD:ADB3808 representing *Phyllomimus* sp14 and ADB6425 representing *Phyllomimus* sp15, were pooled into one MOTU. For the DBTB dataset, RESL analysis generated 42 MOTUs. The members of BOLD:AAY1322 (representing *Gampsocleis sedakovii*, *G. sinensis*, *G. ussuriensis*) were split into 10 MOTUs, and members of BOLD:ADA6837 (representing *G. gratiosa*) were split into three MOTUs.

**Fig. 2:**
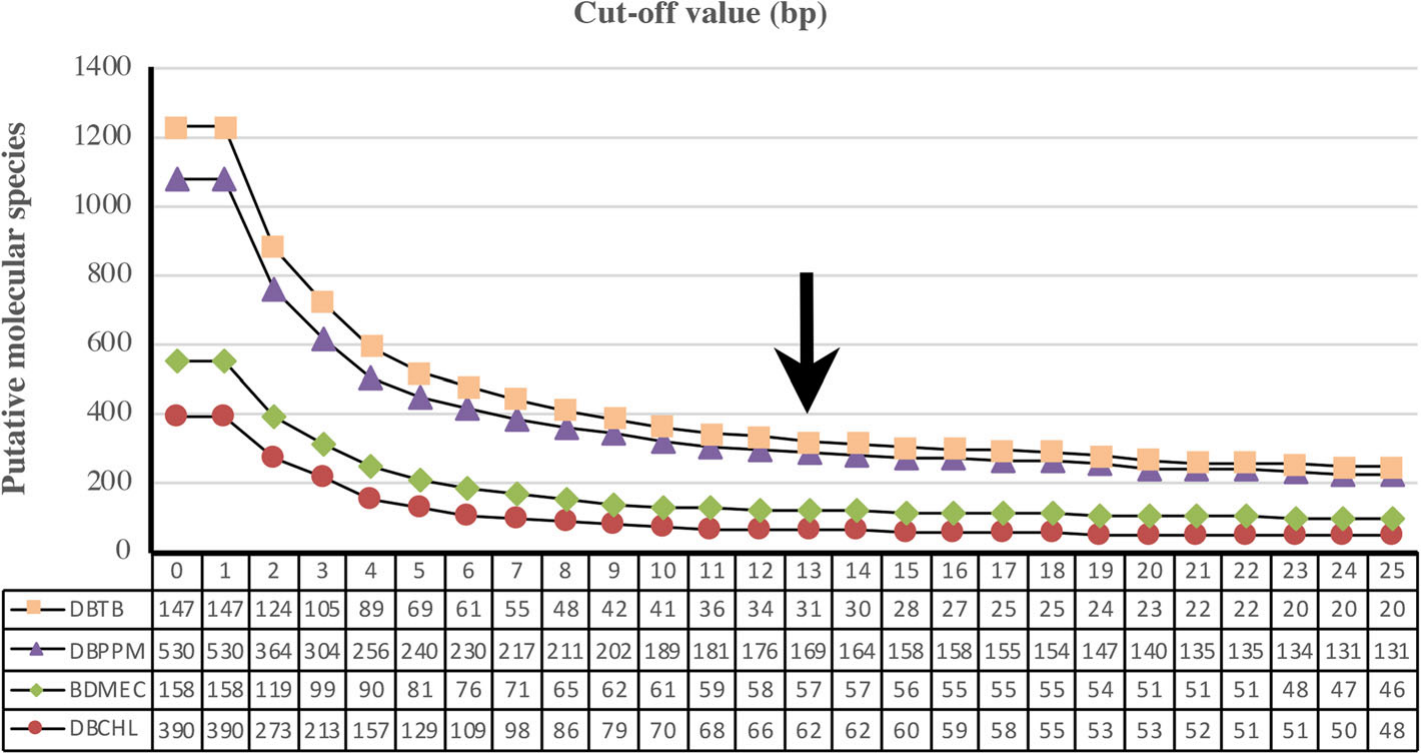
Correspondence between the genetically putative molecular species (MOTUs) number and the cut-off value (bp) generated by jMOTU

**Fig. 3:**
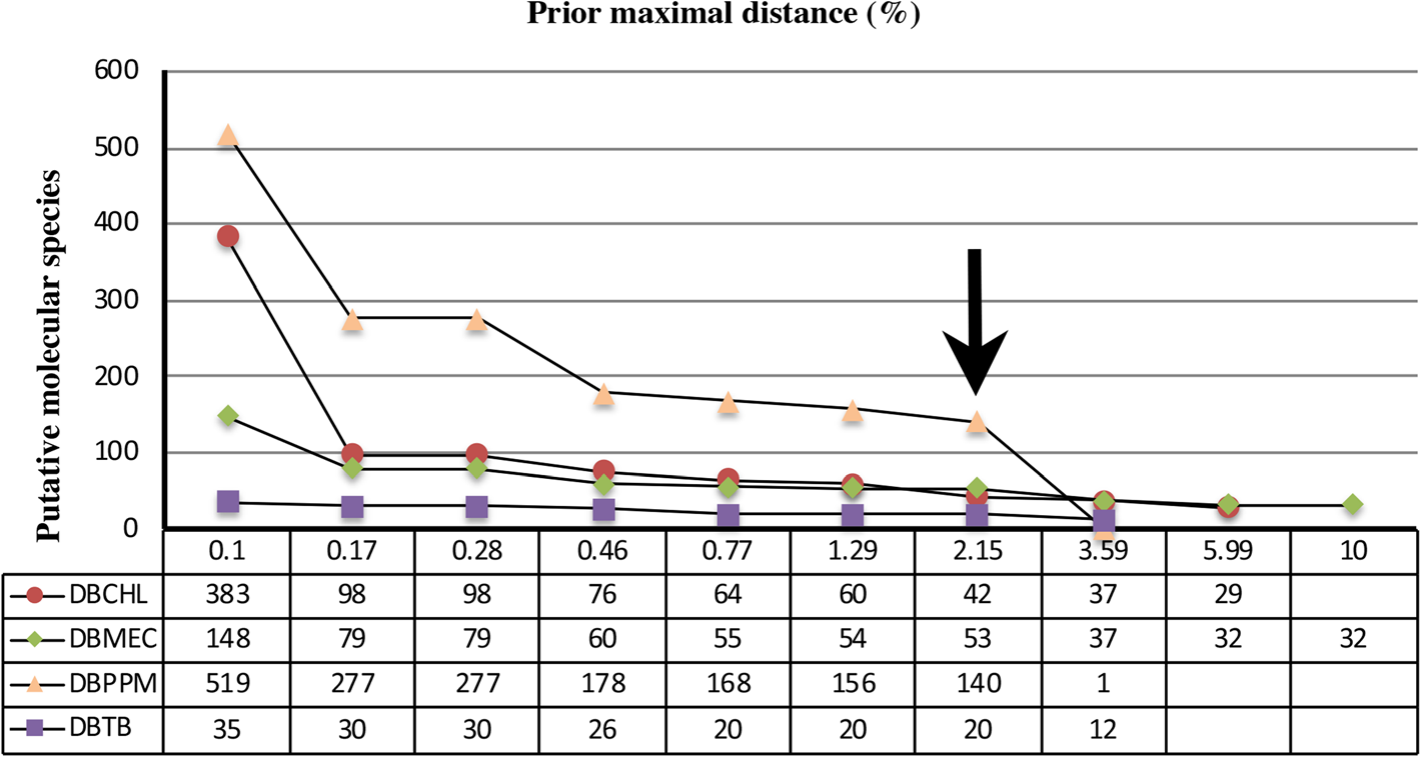
Correspondence between the genetically distinct MOTUs number and prior maximal distance (%) by ABGD based on K2P model

A 2% divergence criterion has been proposed as a general rule-of-thumb for species boundaries with COI-5P [6]. The results of jMOTU analyses at different cutoffs (from 0 to 25 bp) are shown in Fig. 2. A total of 318 MOTUs were determined by a 13 bp (~ 2%) distance cut-off, including 61 MOTUs of DBCHL dataset, 57 MOTUs of DBMEC dataset, 169 MOTUs of DBPPM dataset, and 31 MOTUs of DBTB dataset. ABGD was only based on similarity among sequences, without considering the phylogenetic relationships [19]. The perfect match of ABGD approaching between the initial and the recursive partitions occurred at nucleotide divergence values of 2.15%. The ABGD analyses generated the most conservative results (Table 4) and inferred 255 MOTUs, including 42 MOTUs of DBCHL dataset, 53 MOTUs of DBMEC dataset, 140 MOTUs of DBPPM dataset, and 20 MOTUs of DBTB dataset (Fig. 3).

### Concordance among MOTUs from clustering-based species delimitation methods

Both sGMYC and mGMYC coalescence-based clustering of the specimens were partitioned in the data far more than in all of the other methods (Table 4). The mGMYC analysis was by far the most sensitive of the methods compared, inferring a total of 397 entities, which was slightly higher than sGMYC (*n* = 382). For the DBCHL dataset, the sGMYC analysis identified 87 ML entities (95% confidence interval = 77–95): 71 ML clusters (95% confidence interval = 63–76) and 16 singletons. The mGMYC analysis identified 5 independent switches between speciation and coalescent processes, resulting in 94 ML entities (95% confidence interval = 85–105): 73 ML clusters (95% confidence interval = 67–77) and 21 singletons. For the DBMEC dataset, the sGMYC analysis identified 66 ML entities (95% confidence interval = 48–73): 40 ML clusters (95% confidence interval = 31–40) and 26 singletons. The mGMYC analysis identified 4 independent switches between speciation and coalescent processes, resulting in 60 ML entities (95% confidence interval = 51–76): 36 ML clusters (95% confidence interval = 31–38) and 24 singletons. For the DBPPM dataset, the sGMYC analysis identified 197 ML entities (95% confidence interval = 151–219): 112 ML clusters (95% confidence interval = 93–114) and 85 singletons. The mGMYC analysis identified 6 independent switches between speciation and coalescent processes, resulting in 206 ML entities (95% confidence interval = 179–234): 106 ML clusters (95% confidence interval = 97–113) and 100 singletons. For the DBTB dataset, the sGMYC analysis identified 32 ML entities (95% confidence interval = 20–43): 24 ML clusters (95% confidence interval = 16–29) and 8 singletons. The mGMYC analysis identified 4 independent switches between speciation and coalescent processes, resulting in 37 ML entities (95% confidence interval = 29–59): 23 ML clusters (95% confidence interval = 22–27) and 14 singletons. bPTP analyses inferred 312 MOTUs with wide confidence intervals from MCMC analyses, including 55 MOTUs of DBCHL dataset, 56 MOTUs of DBMEC dataset, 178 MOTUs of DBPPM dataset, and 23 MOTUs of DBTB dataset.

Previous studies have found that in some cases, GMYC could lead to an overestimation of the number of species [57, 58]. GMYC requires prior construction of a ultrametric tree, which does not necessarily reflect the real divergence between species [30]. Alternatively, PTP estimates branching processes using the expected number of substitutions (vs. time in GMYC) and thus utilizes a nonultrametric phylogenetic tree as input [17]. Moreover, in contrast to GMYC, bPTP appeared less sensitive to the sampling regime [17].

### Bidirectional concordance among species delimitation methods with adjusted Wallace coefficients

The adjusted Wallace coefficients were used to compare the bidirectional concordance among species delimitation methods for the identified katydid specimens dataset (Table 8). There were markedly directional results in discriminatory power between the molecular species delimitation methods and morphospecies or across molecular species delimitation methods. For example, the adjusted Wallace coefficient value (0.954) from BIN to morphology meant two specimens within a BIN had a 95.4% chance of belonging to the same morphospecies. In contrast, two specimens within a morphospecies had only a 70.9% chance of belonging to the same BIN. Overall, molecular species delimitation methods had a strong explanatory ability (0.872–0.969) for morphospecies; in contrast, the morphospecies had a generally low explanatory ability (0.391–0.995) for molecular species delimitation results. Both sGMYC and mGMYC were less concordant with morphospecies than other molecular species delimitation results. GMYC inferred a substantially unrealistically high number of katydid MOTUs (Table 4). The morphospecies was best able to explain the results of both ABGD (0.955) and bPTP (0.940). They generally exhibited a more modest ability to explain the molecular results in comparison with all other methods (0.996–1 for ABGD and 0.990–0.998 for bPTP to be explained by all other molecular species delimitation results). The differences among the remaining methods (e.g., BIN, jMOTU, and RESL) in their concordance to the current taxonomy were modest.

**Table 8:** Bidirectional concordance among clustering methods for identified katydids specimens dataset using Adjusted Wallace and 95% CI

	Morphospecies	BIN-species	ABGD	jMOTU	RESL	sGMYC	mGMYC	bPTP
Morphospecies		0.709 (0.683–0.735)	0.955 (0.945–0.965)	0.861 (0.839–0.883)	0.841 (0.827–0.855)	0.391 (0.375–0.408)	0.451 (0.429–0.472)	0.940 (0.929–0.951)
BIN-species	0.954 (0.946–0.963)		1.000 (1.000–1.000)	0.999 (0.999–1.000)	0.999 (0.998–0.999)	0.535 (0.517–0.553)	0.608 (0.583–0.633)	0.998 (0.996–0.999)
ABGD	0.859 (0.846–0.873)	0.668 (0.644–0.693)		0.880 (0.858–0.901)	0.793 (0.779–0.808)	0.367 (0.352–0.382)	0.416 (0.398–0.435)	0.969 (0.959–0.978)
jMOTU	0.881 (0.867–0.894)	0.760 (0.737–0.782)	1.000 (1.000–1.000)		0.874 (0.865–0.883)	0.415 (0.399–0.431)	0.469 (0.448–0.490)	0.995 (0.993–0.996)
RESL	0.953 (0.943–0.963)	0.841 (0.815–0.868)	1.000 (1.000–1.000)	0.969 (0.949–0.990)		0.450 (0.434–0.466)	0.513 (0.492–0.535)	0.998 (0.996–0.999)
sGMYC	0.959 (0.951–0.966)	0.974 (0.964–0.983)	1.000 (1.000–1.000)	0.995 (0.993–0.996)	0.973 (0.963–0.982)		0.916 (0.906–0.927)	0.995 (0.992–0.998)
mGMYC	0.969 (0.964–0.975)	0.972 (0.964–0.980)	0.996 (0.994–0.997)	0.986 (0.983–0.989)	0.974 (0.965–0.982)	0.804 (0.796–0.813)		0.990 (0.987–0.993)
bPTP	0.872 (0.859–0.884)	0.687 (0.663–0.711)	0.998 (0.996–1.000)	0.901 (0.880–0.922)	0.816 (0.803–0.828)	0.376 (0.361–0.392)	0.427 (0.408–0.446)	

Values in parentheses indicate the total number of clusters generated for each analysis. Each value in the table indicates how well the clusters generated by the method indicated by the row label correspond to the clusters yielded by the method indicated in the column label. Each pair of methods is represented by two values in the table

## Discussion

Species was defined as lineages that evolve separately from each other [59]. Determining the species boundaries is one of the central debates in biology [18]. DNA barcoding was widely used for species identification and/or delimitation. Recent research on Central European Orthoptera found that ninety-three of these 122 species (76.2%, including all Ensifera) could be reliably identified using DNA barcodes [37]. In European diving beetles, 36% of multiply sampled species were nonmonophyletic [60]. Our study provides barcode data for 131 identified morphospecies and 148 unidentified BIN-species of Chinese katydids. There was a perfect correspondence between BIN membership and morphospecies in 83 cases, while another 34 species split into more than one BIN. more than one species merges as one BIN or in a combination of merges and splits. The maximum intraspecific distance is less than 3% in 74 of the 109 identified morphospecies (67.89%) represented by multiple individuals. Maximum intraspecific distance is less than 3% in all unidentified BIN-species. Our results revealed a much higher diversity in Chinese katydids than the current taxonomy suggests. There are more katydid species to be described and cryptic lineages within currently recognized species.

### COI-5P barcode and BIN sharing

The causes for barcode and BIN sharing in closely related species include imperfect taxonomy [32], nonfunctional nuclear-encoded mitochondrial pseudogenes (Numts), hybridization, and incomplete lineage sorting [37, 61, 62]. Previous studies have found evidence for frequent hybridization across orthopteran closely related species, such as the genus *Chorthippus* [63], *Aglaothorax* [64], *Tetrix* [65]. COI-5P barcodes sharing was found in two cases, *Gampsocleis sedakovii* (GHF077_16) vs. *G. sinensis* (GHF074_16), as well as *G. sinensis* (RBTC480_16, RBTC1222_16, RBTC1209_16, RBTC1193_16) vs. *G. ussuriensis* (GHF028_16). The barcodes of *G. sedakovii*, *G. sinensis* and *G. ussuriensis* pooled into one discordance BIN (BOLD:AAY1322), but was not supported by our other analyses. The morphological high similarity between *G. sinensis* and *G. ussuriensis*. Another possible reason is that *G. ussuriensis* might in fact synonymised to *G. sinensis*. Meanwhile, *G. sedakovii* and *G. ussuriensis* occur in sympatry over large parts of their distribution ranges. Hybridization in sympatry has resulted the transfer barcodes from *G. sedakovii* to *G. sinensis* and/or *G. ussuriensis*, causing COI-5P barcode and BIN sharing.

Hausmann et al. (2013) suggested that cases of BIN sharing among allopatric, slightly divergent genetic clusters represent recently separated lineages that have recently speciated or are still undergoing genetic differentiation [28]. The bPTP analysis indicated four *Ruspolia* species, *R. dubia*, *R. jezoensis*, *R. liangshanensis*, and *R. yunnana*, pooled into one MOTU. Meanwhile, both RESL and jMOTU analysis indicated *R. dubia*, *R. jezoensis*, and *R. liangshanensis* pooled into one MOTU. Consistent results has also been previously observed in which with regard to *R. jezoensis* synonymised to *R. dubia*, and *R. liangshanensis* may be recently separated from *R. dubia* [36]. *Conocephalus japonicus* is nested within the *C. longipennis* cluster. These species formed very recently indeed, and young species (incomplete lineage sorting) remain within its sister species’ coalescent lead to BIN sharing.

In the last few years, some locally distributed *Xizicus* species have been described. Our analyses found *X. concavilaminus* and *X. kulingensis* pooled into one MOTU (BOLD:ADB3332). *X. laminatus* and *X. tinkhami* pooled into one MOTU (BOLD:ADB5868) except for GMYC analyses. *Xizicus rehni* and *X. howardi* share BOLD:ACD5539, but still exhibit subclusters that separate species at a very low distance. Three discordance BINs (BOLD: ACD5539, ADB3332, and ADB5868) between *Xizicus* species were supported by most analyses. This phenomenon might reflect their relatively recent split or the current taxonomy of *Xizicus* too detailed.

### Morphospecies split into more than one BIN

Our results demonstrate the existence of more than one separate lineage in several katydids with wide geographic distribution range. Some species split into more than one BIN may referred to sister clusters on the barcode trees may representing true potential cryptic diversity [31]. Many species with wide geographic distribution range were placed in either a single BIN or a few, but *Conocephalus maculatus* was outliers, being assigned to 11 BINs. 10 of 11 BINs in *C. maculatus* represented by two or more specimens. Four BINs of *C. maculatus* co-occur at different sites across China, such as BOLD:ACN8107 from Hainan, Yunnan, Guangxi; BOLD:ADB6356 from Xizang, Yunnan; BOLD:ACD2116 from Zhejiang, Jiangxi; BOLD:ADB6002 from Xizang, Yunnan. *C. maculatus* is one of the most widespread species of genus *Conocephalus*, and exhibit one monophyletic cluster. *C. maculatus* is very likely to represent a species complex. Previous research found specimens of one Canadian spiders *Tetragnatha versicolor* were assigned to 20 BINs [9].

The maximum intraspecific distances possessed up to 18.85% in *Conocephalus bidentatus*. All barcodes of *C. bidentatus* exhibit one monophyletic cluster, and clearly distinct subclusters reflected by three BINs. Three BINs within *C. bidentatus* reflect geographic clustering with BOLD:ADB6577 from Fujiang and Zhejiang, BOLD:ADB9596 from Sichuang and BOLD:ADC0531 from Guizhou. Our analyses support *C. bidentatus* split into three MOTUs except for ABGD and mGMYC treating ADB6577 and ADB9596 as one MOTU.

*Xiphidiopsis autumnalis* and *X. gurneyi* form one monophyletic cluster. The two BINs within *X. autumnalis* reflect geographic clustering with ADE1666 from Hainan, ADE1667 from Guangxi. Meanwhile, the four BINs (BOLD:ADB7052, ADE1668, ADE1669, and ADE1670) within *X. gurneyi* reflect different sampled localities. *Xizicus howardi* split into three BINs: ACD5539 from Guangxi, Henan, Hubei, and Zhejiang, ACD5688 from Zhejiang, and ADE3141 from Zhejiang, presumably with a species status. Two or more species are cryptic if they are morphologically similar, biologically distinct, and misclassified as a single species [66]. Cryptic species complexes, in which the component taxa have not diverged morphologically too much, are very difficult to identify, and their discovery is frequently a matter of chance [67]. This phenomenon may suggest possible cryptic species or there are more species to be described.

Conflicts among different species delimitation approaches are very common. Both *Gampsocleis carinata* and *G. gratiosa* were split into two MOTUs except for ABGD analysis. The two BINs (BOLD:ADA6038 and ADA5568) of *Gampsocleis carinata* were predominantly matched to most other clustering methods, which potentially implies detectable intraspecific diversity within *Gampsocleis carinata* and *G. gratiosa*, or the probable existence of more than one species. The species *Tettigonia chinensis* was supported by three clustering methods (ABGD, mGMYC and bPTP). Meanwhile, it was split into two BINs (BOLD:ACD6622 and ACD6623) and was supported by both RESL and jMOTU results.

DNA-based species delimitation may be compromised by limited sampling effort and species rarity, including “singleton” representatives of species, which hampers estimates of intra- versus interspecies evolutionary processes [68]. A broader intraspecific sampling is a critical step for increasing the success of species identification, and a special effort was made to achieve this aim [69]. Previous studies demonstrated broader geographical sampling decreases the barcoding gap between species and hence reduces the accuracy of DNA barcoding [60]. *Ducetia japonica* have been found are distributed over a huge area extending from Pakistan in the West to the Solomon Islands in the East and from Northern China in the North to northern Australia in the South [70]. We have a broader sampling of *Ducetia japonica* distribution in China. Our results demonstrate the existence of three separate lineages (BOLD:ACD7324, ADB6191, and ACE7214) in *D. japonica*. The different song types indicated clearly that *D. japonica* as presently understood is not a homogeneous, extremely widespread species, but a complex of several distinct species [70].

The nonfunctional nuclear-encoded mitochondrial pseudogenes (Numts) are a potential source of barcoding error [31]. Previous studies have showed that Numts had devastating influence on DNA barcoding results and were very hard to detect [71–73]. Our analyses supported *Euxiphidiopsis capricercus* split into two BINs (BOLD:ADB5001 and ADE2467) except for GMYC. Only one specimen (HLXX121–16) correspond to BOLD:ADE2467, and differed from the remaining *E. capricercus* specimens by 27.45%. *E. capricercus* HLXX121–16 as the NN of *Gampsocleis gratiosa*, the distance only 2.49%. Note that excluding *E. capricercus* BOLD:ADE2467 results the intraspecific distances (1.23%) significant decrease. BOLD:ADE2467 far from the *Euxiphidiopsis* cluster on the NJ-K2P tree. The sequence divergence within *E. capricercus* and geographical distance is correlated significantly. *E. capricercus* HLXX121–16 (BOLD:ADE2467).

## Conclusions

No universal barcode gap was observed in our four data sets. There are moderately variable results from different delimitation methods. Our research supported the contention of Ortiz and Francke (2016) contention that combining evidence from multiple delimitation methods obtains better-supported results [18]. To diminish the probability of species under- or overestimation solutions, we determined separate MOTUs that were recovered in at least four of the seven species delimitation analyses. Excluding singletons (22 identified species, 125 BINs), we recognized 62 robustly supported identified morphospecies, and 166 BIN-species. The GMYC model exhibited a characteristic “overestimating” solutions.

If most MOTU splits detected in this study reflect cryptic/undescribed taxa, the true species count for Chinese katydids could be a large proportion higher than currently recognized. Moreover, only less than 20% species (50 of 279) were represented by not less than 10 specimens, and expanded sample sizes might reveal more barcode splits. Here, we refrain from taxonomic descriptions, as this requires a thorough morphological and taxonomic study for each putative taxon. It is also important to note that there could be noise in our results, potentially due to considerable unidentified specimens. Nevertheless, our results support COI-5P efficacy for rapid delimitation of katydid species and for indicating likely cryptic/undescribed species for further exploration.

## Additional files

Additional file 1: **Table S1.** Additional 34 COI-5P sequences that were mined from GenBank. (XLSX 23 kb)

Additional file 2: **Table S2.** The distance within-species and to its nearest neighbor (NN). (XLSX 21 kb)

Additional file 3: Comparison of the species delimitation results of Chinese katydids based on an analysis of 390 unique COI-5P haplotypes of the DBCHL dataset. A midpoint-rooted NJ-K2P tree was implemented in MEGA 7.0. Terminals were labeled with Sequence/Process ID, Species identifications, plus BIN. * indicated a haplotype representing more than one specimen. ** indicated a haplotype shared by more than one species. On the right: summary of putative species delimitation drawn by BINs, RESL, jMOTU, ABGD, sGMYC, mGMYC and bPTP (one column per method). Black codes represented putative MOTUs defined by at least four of the seven species delimitation methods. Grey codes represented MOTUs defined by less than four of the seven species delimitation methods. Other color codes for each column represented clustering together as a single MOTU. (TIF 8640 kb)

Additional file 4: Comparison of the species delimitation results of Chinese katydids based on an analysis of 158 unique COI-5P haplotypes of the DBMEC dataset. A midpoint-rooted NJ-K2P tree was implemented in MEGA 7.0. Terminals were labeled with Sequence/Process ID, Species identifications, plus BIN. * indicated a haplotype representing more than one specimen. ** indicated a haplotype shared by more than one species. On the right: summary of putative species delimitation drawn by BINs, RESL, jMOTU, ABGD, sGMYC, mGMYC and bPTP (one column per method). Black codes represented putative MOTUs defined by at least four of the seven species delimitation methods. Grey codes represented MOTUs defined by less than four of the seven species delimitation methods. Other color codes for each column represented clustering together as a single MOTU. (TIF 3270 kb)

Additional file 5: Comparison of the species delimitation results of Chinese katydids based on an analysis of 530 unique COI-5P haplotypes of the DBPPM dataset. A midpoint-rooted NJ-K2P tree was implemented in MEGA 7.0. Terminals were labeled with Sequence/Process ID, Species identifications, plus BIN. * indicated a haplotype representing more than one specimen. ** indicated a haplotype shared by more than one species. On the right: summary of putative species delimitation drawn by BINs, RESL, jMOTU, ABGD, sGMYC, mGMYC and bPTP (one column per method). Black codes represented putative MOTUs defined by at least four of the seven species delimitation methods. Grey codes represented MOTUs defined by less than four of the seven species delimitation methods. Other color codes for each column represented clustering together as a single MOTU (TIF 1150 kb)

Additional file 6: Comparison of the species delimitation results of Chinese katydids based on an analysis of 147 unique COI-5P haplotypes of the DBTB dataset. A midpoint-rooted NJ-K2P tree was implemented in MEGA 7.0. Terminals were labeled with Sequence/Process ID, Species identifications, plus BIN. * indicated a haplotype representing more than one specimen. ** indicated a haplotype shared by more than one species. On the right: summary of putative species delimitation drawn by BINs, RESL, jMOTU, ABGD, sGMYC, mGMYC and bPTP (one column per method). Black codes represented putative MOTUs defined by at least four of the seven species delimitation methods. Grey codes represented MOTUs defined by less than four of the seven species delimitation methods. Other color codes for each column represented clustering together as a single MOTU. (TIF 3020 kb)
