# Supplementary figures and images for: Singleton molecular species delimitation based on COI-5P barcode sequences revealed high cryptic/undescribed diversity for Chinese katydids (Orthoptera: Tettigoniidae)
Source: BMC Evol Biol. 2019 Mar 14;19:79. doi: 10.1186/s12862-019-1404-5 (PMC6419471; doi:10.1186/s12862-019-1404-5)

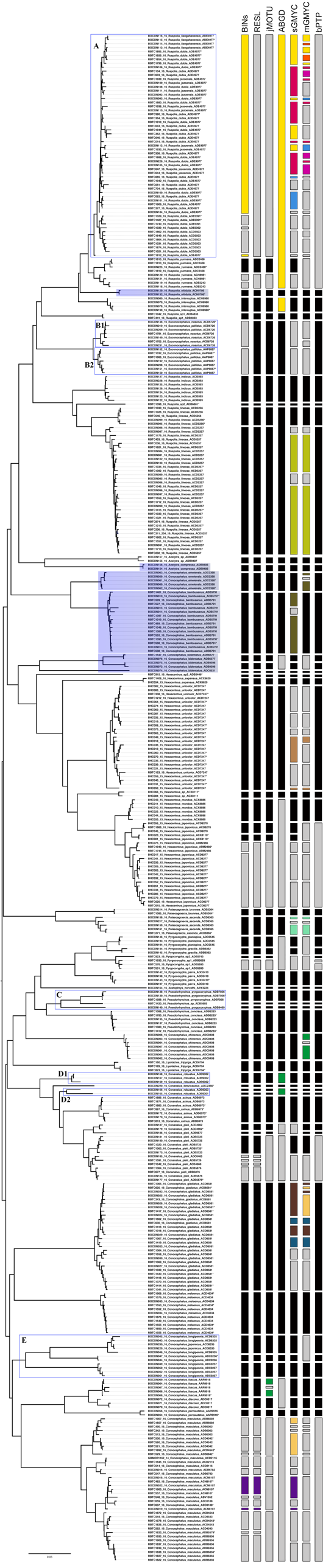

Supplement: Supplementary file 3 — Comparison of the species delimitation results of Chinese katydids based on an analysis of 390 unique COI-5P haplotypes of the DBCHL dataset. A midpoint-rooted NJ-K2P tree was implemented in MEGA 7.0. Terminals were labeled with Sequence/Process ID, Species identifications, plus BIN. * indicated a haplotype representing more than one specimen. ** indicated a haplotype shared by more than one species. On the right: summary of putative species delimitation drawn by BINs, RESL, jMOTU, ABGD, sGMYC, mGMYC and bPTP (one column per method). Black codes represented putative MOTUs defined by at least four of the seven species delimitation methods. Grey codes represented MOTUs defined by less than four of the seven species delimitation methods. Other color codes for each column represented clustering together as a single MOTU. (TIF 8640 kb) [file 12862_2019_1404_MOESM3_ESM.tif]

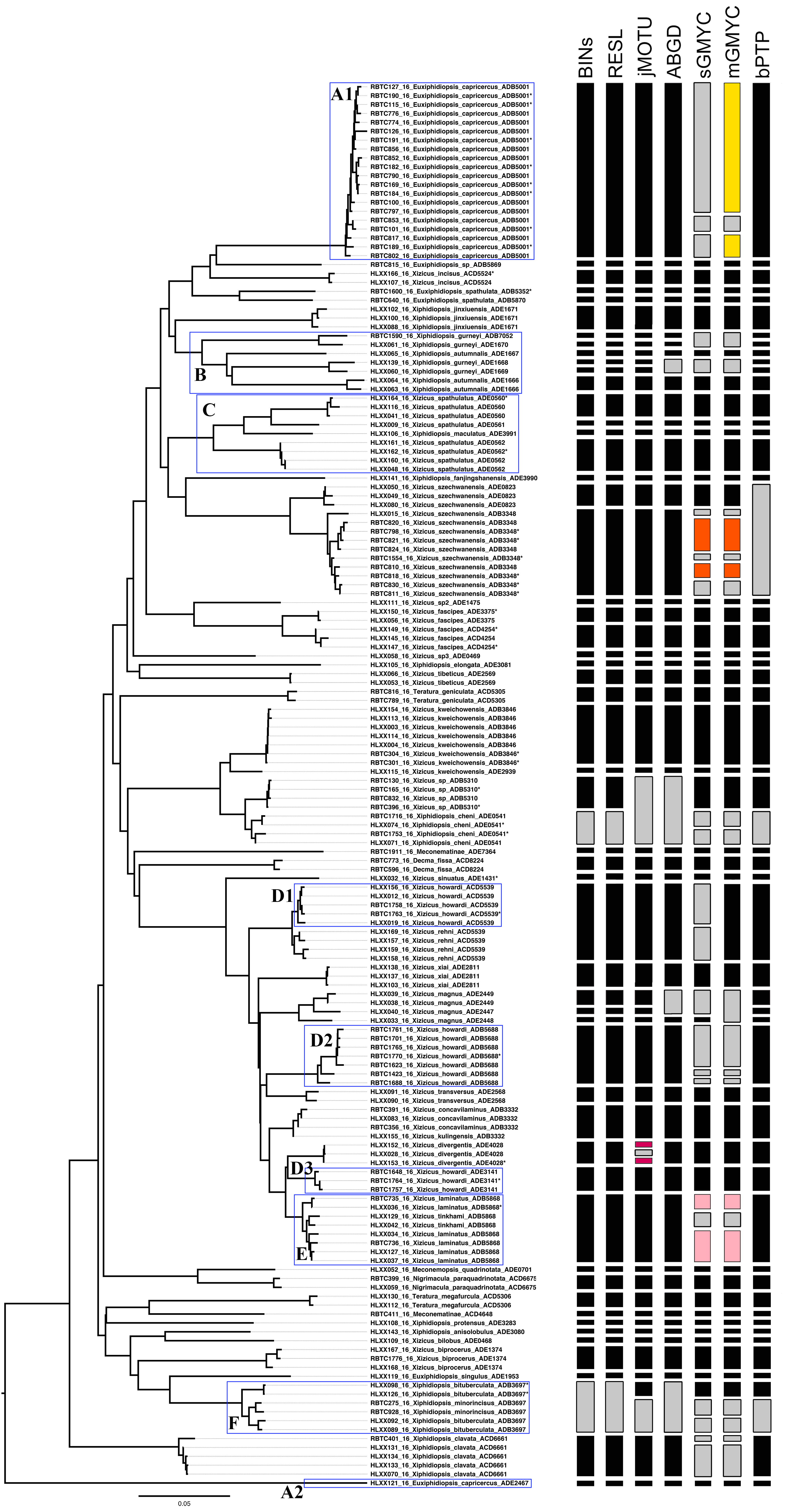

Supplement: Supplementary file 4 — Comparison of the species delimitation results of Chinese katydids based on an analysis of 158 unique COI-5P haplotypes of the DBMEC dataset. A midpoint-rooted NJ-K2P tree was implemented in MEGA 7.0. Terminals were labeled with Sequence/Process ID, Species identifications, plus BIN. * indicated a haplotype representing more than one specimen. ** indicated a haplotype shared by more than one species. On the right: summary of putative species delimitation drawn by BINs, RESL, jMOTU, ABGD, sGMYC, mGMYC and bPTP (one column per method). Black codes represented putative MOTUs defined by at least four of the seven species delimitation methods. Grey codes represented MOTUs defined by less than four of the seven species delimitation methods. Other color codes for each column represented clustering together as a single MOTU. (TIF 3270 kb) [file 12862_2019_1404_MOESM4_ESM.tif]

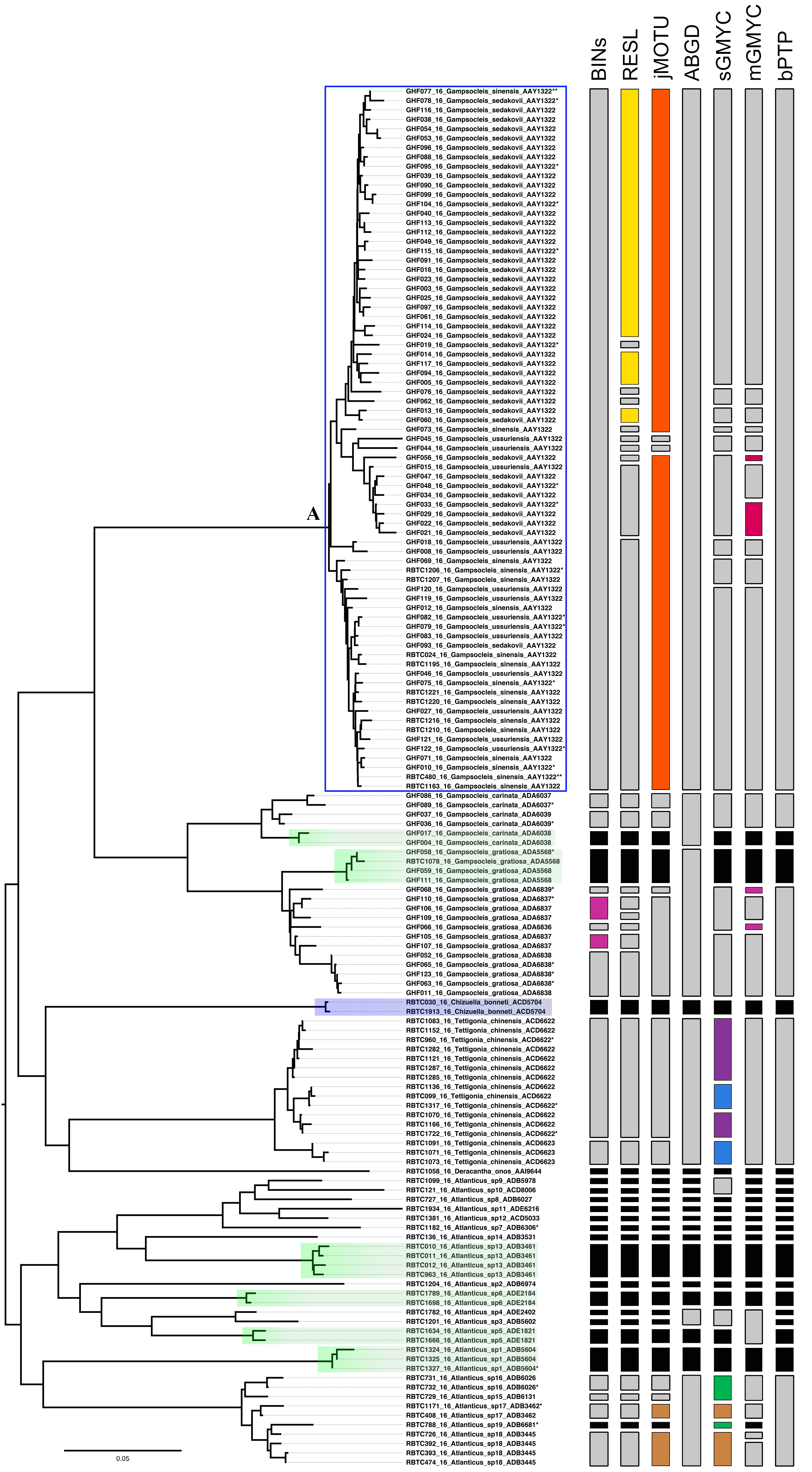

Supplement: Supplementary file 6 — Comparison of the species delimitation results of Chinese katydids based on an analysis of 147 unique COI-5P haplotypes of the DBTB dataset. A midpoint-rooted NJ-K2P tree was implemented in MEGA 7.0. Terminals were labeled with Sequence/Process ID, Species identifications, plus BIN. * indicated a haplotype representing more than one specimen. ** indicated a haplotype shared by more than one species. On the right: summary of putative species delimitation drawn by BINs, RESL, jMOTU, ABGD, sGMYC, mGMYC and bPTP (one column per method). Black codes represented putative MOTUs defined by at least four of the seven species delimitation methods. Grey codes represented MOTUs defined by less than four of the seven species delimitation methods. Other color codes for each column represented clustering together as a single MOTU. (TIF 3020 kb) [file 12862_2019_1404_MOESM6_ESM.tif]
